# Pattern of sympathetic vasomotor activity in a model of hypertension induced by nitric oxide synthase blockade

**DOI:** 10.14814/phy2.14183

**Published:** 2019-07-19

**Authors:** Lysien I. Zambrano, Roberto B. Pontes, Michelle L. Garcia, Erika E. Nishi, Fernando N. Nogueira, Elisa M. S. Higa, Juliana G. Cespedes, Cassia T. Bergamaschi, Ruy R. Campos

**Affiliations:** ^1^ Department of Physiology, Cardiovascular Division Escola Paulista de Medicina‐Universidade Federal de São Paulo São Paulo Brazil; ^2^ Department of Morphology, School of Medical Sciences National Autonomous University of Honduras Tegucigalpa Honduras; ^3^ Departamento de Biomateriais e Biologia Oral, Faculdade de Odontologia Universidade de São Paulo São Paulo Brazil; ^4^ Nephrology Division Escola Paulista de Medicina Universidade Federal de São Paulo São Paulo Brazil; ^5^ Institute of Science and Technology Universidade Federal de São Paulo São Jose dos Campos Brazil

**Keywords:** Baroreflex sensitivity, hypertension, nitric oxide, sympathetic vasomotor activity

## Abstract

We aimed to investigate the effects of nitric oxide (NO) synthesis inhibition by NO synthase inhibitor N‐nitro‐L‐arginine‐methyl ester (L‐NAME) treatment on the sympathetic vasomotor nerve activity (SNA) on two sympathetic vasomotor nerves, the renal and splanchnic. NO plasma level and systemic oxidative stress were assessed. Hypertension was induced by L‐NAME (20 mg/kg per day, by gavage, for seven consecutive days) in male Wistar rats. At the end of the treatment, blood pressure, heart rate, arterial baroreflex sensitivity, renal SNA (rSNA), and splanchnic SNA (sSNA) were assessed in urethane anesthetized rats. L‐NAME‐treated rats presented increased blood pressure (152 ± 2 mmHg, *n* = 17) compared to the control group (101 ± 2 mmHg, *n* = 15). Both rSNA (147 ± 10, *n* = 15 vs. 114 ± 5 Spikes/s, *n* = 9) and sSNA (137 ± 13, *n* = 14 vs. 74 ± 13 spikes/s, *n* = 9) were significantly increased in the L‐NAME‐treated compared to the control group. A differential response on baroreflex sensitivity was found, with a significant reduction for rSNA but not for sSNA arterial baroreceptor sensitivity in L‐NAME‐treated rats. The adjusted regression model revealed that the reduction of systemic NO levels partially explains the variation in sSNA and blood pressure, but not rSNA. Taken together, our data show that hypertension induced by NO synthase blockade is characterized by increased SNA to the rSNA and sSNA. In addition, we found that the rats that had the greatest reduction in NO levels in plasma by L‐NAME were those that developed higher blood pressure levels. The reduction in the NO level partially explains the variations in sSNA but not in rSNA.

## Introduction

Nitric oxide (NO) has been described as an important cellular signaling molecule involved in several physiological and pathological conditions (Sakuma et al., 1992; Hirai et al., 1995). Considering that NO is a powerful vasodilator, changes in NO actions on blood vessels have been associated with the development and/or maintenance of increased vascular resistance, arterial hypertension, and cardiovascular and renal dysfunction (Sander et al., 1997; Fadel, 2017).

NO is produced via oxidation of L‐arginine by nitric oxide synthase (NOS) in the vasculature. This is a classic pathway of endothelium‐dependent vasodilation involved in the local control of blood flow and, consequently, the systemic arterial blood pressure (Moncada and Higgs, 2006). In fact, either the acute or chronic inhibition of NO synthesis by intravenous administration of N‐nitro‐L‐arginine methyl ester (L‐NAME) induces a sustained increase in blood pressure (Rees et al., 1989; Ribeiro et al., 1992). However, the mechanisms underlying the NO deficiency‐induced hypertension are not fully understood. Initially, the increase in blood pressure was exclusively attributed to a consequence of withdrawal of the vasodilator actions of NO, and therefore, studies were focused on its vascular actions with little consideration for a role of NO in other tissues (Rees et al., 1989; Vallance et al., 1989; Aisaka et al., 1991; Guix et al., 2005). Currently, considering the pleiotropic actions of NO (e.g., its involvement in the control of brain functions), another mechanism, such as the sympathetic vasomotor activation, has been noted as an important mechanism underlying the hypertension induced by NO synthesis blockade (Bergamaschi et al., 1999; Peotta et al., 2007; Esler et al., 2010).

There is strong evidence that suggests that the brain‐derived NO synthesis is an important mechanism that potently suppresses sympathetic vasomotor activity (Sander et al., 1997; Sander and Victor, 1999; Zucker et al., 2004). Recently, it has been reported that NO synthesis inhibition significantly increased glutamatergic neurotransmission within the rostral ventrolateral medulla (RVLM), an important brain region involved in cardiovascular control, suggesting that NO actions in the brain may be essential to regulate the level of sympathetic vasomotor activity and thus the systemic arterial blood pressure (Machado et al., 2016).

It has also been suggested that the sympathetic hyperactivity following NO synthesis inhibition may appear at the late phase, but not at the initial phase of hypertension development (Cunha et al., 1993; Bergamaschi et al., 1999). One hypothesis that may explain this phenomenon is that in the initial phase of NO synthesis inhibition, the intense baroreceptor activation induced by the acute increase in blood pressure plays an important role against the increase in sympathetic vasomotor activity (Peotta et al., 2007; Borges et al., 2008). This hypothesis was supported by the finding that in arterial baroreceptor denervated animals, sympathetic hyperactivity appears even at the initial phase of NO synthesis inhibition (Malpas et al., 2006). Furthermore, in humans, skin sympathetic vasomotor activity, which is not under baroreceptor control, was significantly increased by systemic NOS inhibition in a time‐dependent manner, supporting the idea that NO inhibition triggers sympathoexcitation leading to hypertension (Young et al., 2009).

One possible mechanism triggering sympathoexcitation following NO inhibition is the activation of the renin‐angiotensin system, which is a potent generator of reactive oxygen species (Aisaka et al., 1991; Crowley, 2014). In fact, angiotensin II is able to activate the NADPH oxidase enzyme leading to an increase in intracellular superoxide anion (O^‐^_2_) formation; this anion functions in the brain as a powerful cellular signaling mechanism leading to sympathoexcitation (Oliveira‐Sales et al., 2009; Collister et al., 2016). In contrast, recently it was reported that L‐NAME‐induced hypertension is not dependent on increased Angiotensin II levels (Simko et al., 2018).

Despite the large amount of evidence, the question of whether the sympathetic vasomotor activity is increased following NO synthesis inhibition and contributes to hypertension is still a matter of discussion. Dos Santos and co‐workers described that there is no increase in renal sympathetic nerve activity after 2 and 14 days of L‐NAME treatment in conscious rats (dos Santos et al., 2010). In contrast, it has been demonstrated that total renal denervation protects against hypertension induced by L‐NAME (Matsuoka et al., 1994). The different results obtained by these studies could be either a consequence of variations in NO bioavailability or a differential response induced by the blockade of distinct NO synthase isoforms (endothelial, neuronal, inducible, and mitochondrial). Therefore, differential actions of NO blockade on specific sympathetic targets could not be excluded. Thus, in the present study, we aimed to investigate the effects of NO synthesis inhibition by L‐NAME treatment on postganglionic sympathetic vasomotor nerve activity in the renal and splanchnic sympathetic vasomotor nerves. Considering that the bioavailability of NO and oxidative stress may be important factors involved in the autonomic and cardiovascular effects induced by NO synthesis inhibition, the plasma level of NO and systemic oxidative stress was assessed in both control and L‐NAME‐treated rats.

## Materials and Methods

### Animals and ethics approval

All experimental procedures performed in this study were conducted under the guidelines recommended by the National Institutes of Health and approved by the Ethics in Research Committee of the Universidade Federal de São Paulo ‐ Escola Paulista de Medicina (process No. 2450240314/2014). Male Wistar rats (250–300 g) were housed in groups of four in standard polypropylene cages; the room was maintained at 22 ± 1°C with a 12:12 h light‐dark cycle (lights on at 7 am), and the rats were allowed free access to food and water. Rats were distributed into two experimental groups: control (CTL) and hypertensive (L‐NAME).

Hypertension was induced by the inhibition of NO synthase with L‐NAME (Sigma‐Aldrich, St. Louis, MO). Animals were treated orally by gavage with L‐NAME (20 mg/kg/day) for seven consecutive days. The dose was chosen based on previous experiments from our group (Biancardi et al., 2007).

### Analysis of cardiovascular function, splanchnic, and renal baroreceptor reflex sensitivity in urethane‐anesthetized rats

After the last L‐NAME treatment (day 7) the rats were anesthetized with ketamine and xylazine (100 mg/kg and 10 mg/kg IP, respectively) for femoral artery and vein catheterization. After surgical recovery (**≥**24 h), blood sample (0.5 mL) was carefully collected through the arterial catheter in the conscious rats for posterior analysis (NO measurement). Pulsatile arterial blood pressure of conscious rats was recorded using a blood pressure signal amplifier (PowerLab System, ADInstruments, Sydney, Australia). The average values of baseline mean arterial pressure (MAP) and heart rate (HR) were obtained from the pulsatile arterial blood pressure wave by continuous recording over 15–20 min. Following stable baseline recordings, rats were slowly anesthetized with urethane (1.4 g/kg, iv; Sigma‐Aldrich, St. Louis, MO) to avoid any change in MAP. It has been described that urethane does not significantly change the gain of baroreceptor reflex sensitivity of SNA (Shimokawa et al., 1998). After tracheostomy, a retroperitoneal incision was then performed to expose both renal and splanchnic nerves. For the renal (rSNA) and splanchnic sympathetic nerve activity (sSNA) recordings, the left renal and splanchnic nerves were placed on bipolar silver electrodes, and once the conditions for nerves recording were established, the nerves and electrodes were covered with paraffin oil. The signal from the renal and splanchnic nerves was displayed on an oscilloscope (TDS 220; Tektronix, Portland, OR), and the nerve activities were amplified (gain 20K, Neurolog; Digitimer, Welwyn Garden City, UK) and filtered by a band‐pass filter (100–1000 Hz). Data analysis was then performed using the PowerLab data acquisition system (ADInstruments, Australia) with a sampling rate of 2000 Hz. At the end of the experiments, the background noise level of the SNA was determined following hexamethonium bromide administration (30 mg/kg, iv; Sigma‐Aldrich). The sSNA and rSNA were rectified online, integrated from the raw data obtained for each heart period, and expressed as spikes/s. In addition, the neural activity was analyzed offline using the appropriate software (Spike Histogram; ADInstruments, Australia), as previously described (Oliveira‐Sales et al., 2009).

For the analysis of arterial baroreceptor control of sSNA and rSNA, blood pressure was altered by ramp infusion of phenylephrine (10 µg in 0.1 mL, iv) or sodium nitroprusside (20 µg in 0.1 mL, iv) to raise or lower blood pressure by approximately 40 mmHg over 60 s, respectively. Phenylephrine and sodium nitroprusside (Sigma‐Aldrich, St. Louis, MO) were infused with a 3‐ml syringe mounted on a syringe pump (KD Scientific) as described elsewhere (Farah et al., 1999). The baroreflex sensitivity was evaluated by the reflex changes of rSNA or sSNA in response to every 5 mmHg of MAP change (from 5 to 40 mmHg). The slope analysis represents the baroreflex gain (ΔrSNA/ΔMAP or ΔsSNA/ΔMAP) from each individual animal and was expressed as spikes/s/mmHg. As steeper is the regression line, higher is the baroreflex sensitivity.

### Nitric oxide determination

The serum was obtained by centrifugation (1620 × *g* for 15 min; Eppendorf 5810, AG, Hamburg, Germany), and NO concentration was determined by the Griess colorimetric method as previously described (Green et al., 1982).

### Biochemical analyses

In another set of experiments, blood samples (1 mL) were collected in CLT (*n* = 6) and L‐NAME‐treated rats (*n* = 6), and superoxide dismutase (SOD) and glutathione peroxidase (GPx) enzyme activities in the serum were determined using RANSOD and RANSEL kits (Randox Laboratories, Crumlin, UK), respectively. Catalase (CAT) activity was assayed as previously described (Aebi, 1984). Lipid peroxidation was estimated based on serum levels of malondialdehyde (MDA) (Karatas et al., 2002). The protein concentration was measured by the Bradford method.

### Statistical analyses

Results are expressed as the mean ± standard error of the mean (SEM). First, the data were evaluated by an unpaired Student’s *t*‐test using the R program (R core Team, 2016). Next, the relationships between MAP and rSNA as well as MAP and sSNA were analyzed; MAP and plasma levels of NO were compared among both the control (CLT) and the L‐NAME rats using linear regression models. The assumptions of normality and homogeneity of variance were verified by the Shapiro‐Wilk and Breusch–Pagan tests, respectively. In the third step, the correlations were assessed only for the L‐NAME‐treated group. The level of statistical significance was defined as *P < *0.05.

## Results

### Cardiovascular parameters and serum NO

**Figure 1:**
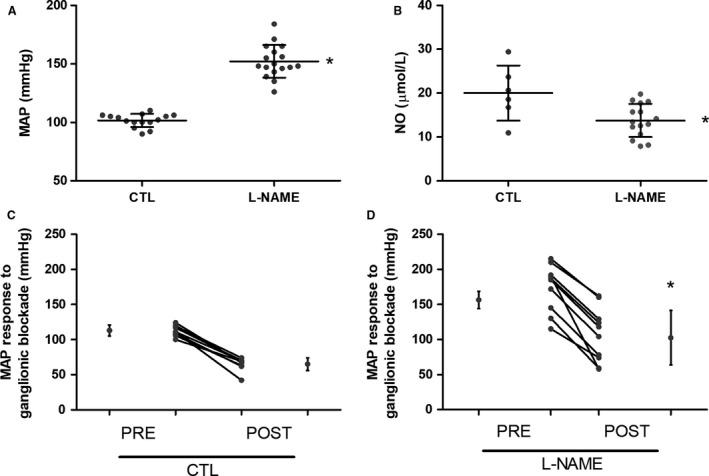
Baseline values of MAP in urethane anesthetized rats, serum NO concentration (B), and MAP changes following ganglionic inhibition with hexamethonium bromide administration (30 mg/Kg, iv) in CTL (C) and L‐NAME‐treated (D) groups. *P < 0.05 compared to CTL group.

L‐NAME‐treated rats presented a significant increase in MAP (152 ± 2 mmHg, *n* = 17) compared with control (CTL) rats (101 ± 1 mmHg, *n* = 15; Fig. 1A); however, there were no significant differences in the baseline HR. Serum NO measurements revealed a significant reduction in L‐NAME (13.76 ± 1.0 µmol/L, *n* = 15) compared to CTL group (20.0 ± 2.6 µmol/L, *n* = 6; Fig. 1B). Ganglionic blockade with hexamethonium induced a significantly greater decrease in MAP in the L‐NAME‐treated rats (from 152 ± 3 mmHg to 106 ± 12 *n* = 9; Fig. 1D) than in the CTL group (101 ± 1.4 mmHg to 66 ± 10 mmHg *n* = 10; Fig. 1C).

### Basal and reflex control of renal sympathetic nerve activity (rSNA) and splanchnic sympathetic nerve activity (sSNA) by the arterial baroreceptors

**Figure 2:**
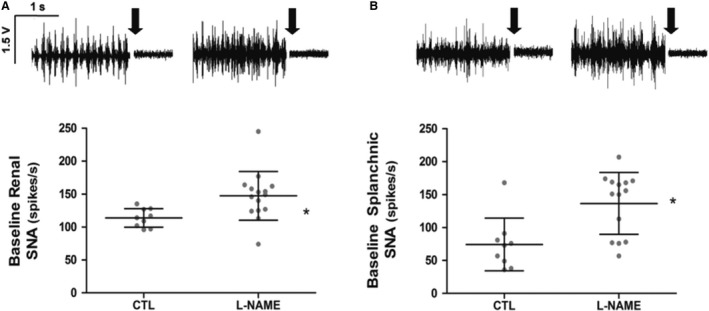
Baseline values of renal sympathetic nerve activity (rSNA) (A) and splanchnic (sSNA) (B) in CTL and L‐NAME‐treated groups. Upper panels show representative tracings of SNA for each group. *P < 0.05 compared with the CTL. Arrows show intravenous hexamethonium administration.

The L‐NAME group showed a significant increase in baseline values of rSNA (Fig. 2A; L‐NAME 147 ± 10 *n* = 15; CLT 114 ± 5 spikes/s, *n* = 9) and sSNA (Fig. 2B; L‐NAME, 137 ± 13, *n* = 14; CLT, 74 ± 13 spikes/s, *n* = 09).

**Figure 3:**
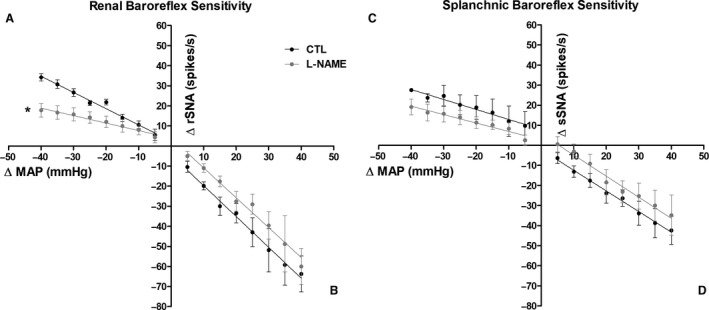
Arterial baroreflex sensitivity for sympathetic nerve activity (SNA) reflex responses to the kidney (rSNA) (A‐B) and splanchnic (sSNA) (C‐D) nerves. Reflex increase in rSNA (A) and sSNA (C) in response to the decrease in mean arterial pressure (MAP) produced by ramp infusion of sodium nitroprusside (20 μg in 0.1 mL over 60 s, iv) and reflex decrease in rSNA (B) and sSNA (D) in response to the increase in MAP produced by ramp infusion of phenylephrine (10 μg in 0.1 mL over 60 s, iv) in CTL and L‐NAME‐treated groups. *P < 0.05 compared with the CTL.

The arterial baroreceptor reflex function for rSNA (Fig. 3A and B) and sSNA (Fig. 3C and D) shows a differential response on baroreflex sensitivity in L‐NAME group. A significant reduction in baroreflex sensitivity for rSNA (Fig. 3A) but not for sSNA (Fig. 3C) during unloading of arterial baroreceptor was observed. Table 1 shows the values of baroreflex gain for the L‐NAME‐treated and CLT groups.

**Table 1:** Baroreflex gain assessed by reflex changes in renal and splanchnic sympathetic vasomotor activity (rSNA and sSNA, respectively) in response to mean arterial pressure (MAP) variations induced by systemic administration of vasoactive drugs.

Group	rSNA reflex increase (spikes/s/mmHg)	rSNA reflex decrease (spikes/s/mmHg)	sSNA reflex increase (spikes/s/mmHg)	sSNA reflex decrease (spikes/s/mmHg)
CTL	−0.807 ± 0.07 *n* = 9	−1.536 ± 0.33 *n* = 9	−0.497 ± 0.19 *n* = 9	−1.028 ± 0.24 *n* = 9
L‐NAME	−0.372 ± 0.13 *n* = 9*	−1.370 ± 0.25 *n* = 8	−0.614 ± 0.20 *n* = 9	−1.104 ± 0.16 *n* = 8

Values are expressed as mean ± standard error of the mean.

* *P* < 0.05 compared with the CTL.

### Correlation between NO, MAP, sSNA, and rSNA

**Figure 4:**
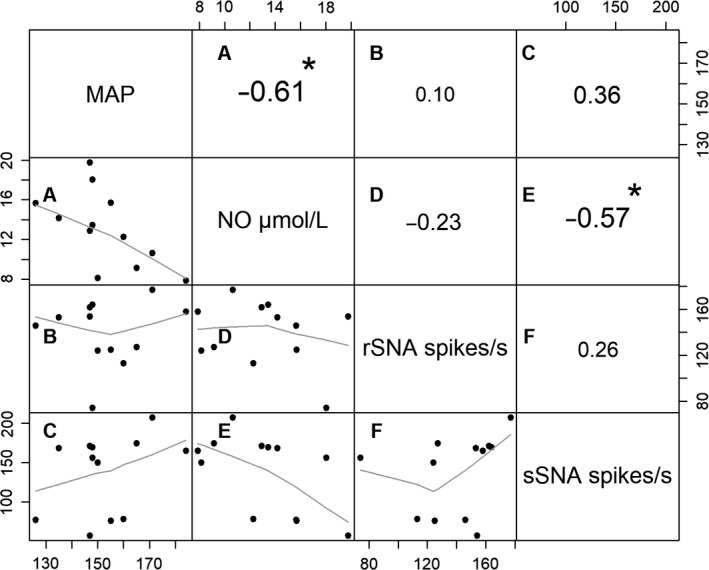
Matrix of correlations between the variables MAP, NO, rSNA, and sSNA measured in real terms. Test for association between MAP and serum concentration of NO (A); test for association between MAP and rSNA (B); test for association between MAP and sSNA (C); test for association between serum concentration of NO and rSNA (D); test for association between serum concentration of NO and sSNA (E); test for association between rSNA and sSNA (F). *P < 0.05.

The matrix of correlations between the variables, MAP, NO, rSNA, and sSNA, is shown in Figure 4. The values of the correlations were measured in real terms and the statistical significance is interpreted in absolute values. The higher its graphical representation, the greater its significance. Considering *P* < 0.05, the correlations between NO versus MAP and NO versus sSNA were significantly different from zero.

**Figure 5:**
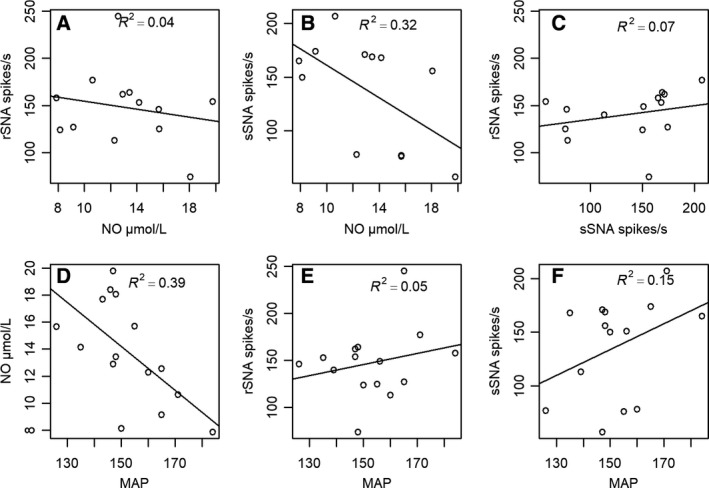
Correlation using linear regression model between splanchnic sympathetic nerve activity (sSNA) and renal sympathetic nerve activity (rSNA) (A). Correlation between serum concentration of nitric oxide (NO) and sSNA (B). Correlation between plasma concentration of nitric oxide (NO) and rSNA (C).Correlation between MAP and nitric oxide (NO) serum concentration (D). Correlation between MAP and rSNA (E). Correlation between MAP and sSNA (F).

In Figure 5 **,** the correlations for the L‐NAME‐treated group are presented using linear regression models. For the correlation between sSNA and plasma levels of NO**,** the adjusted regression model revealed that the reduction in the NO level partially explains the variation in sSNA and, in this case, showed a significant correlation between variables, with *R* ^2^ = 0.32 (Fig. 5B). However, the correlation between rSNA and plasma levels of NO did not explain the variation in rSNA; considering the result of the *R* ^2^ = 0.04, the correlation between the variables was not statistically significant (Fig. 5A).

No correlation, however, was found between sSNA and rSNA (*R* ^2^ = 0.07; Fig. 5C). The comparison between the plasma levels of NO and MAP indicated that the correlation between variables was significant in the L‐NAME group with *R* ^2^ = 0.39 (Fig. 5D), thus, the rats that had the greatest reduction in NO levels in plasma by L‐NAME were those that developed higher blood pressure levels. However, no significant correlation was found between MAP and rSNA (*R* ^2^ = 0.05; Fig. 5E) or between MAP and sSNA (*R* ^2^ = 0.15; Fig. 5F).

### Oxidative stress markers and antioxidant enzyme activity

**Figure 6:**
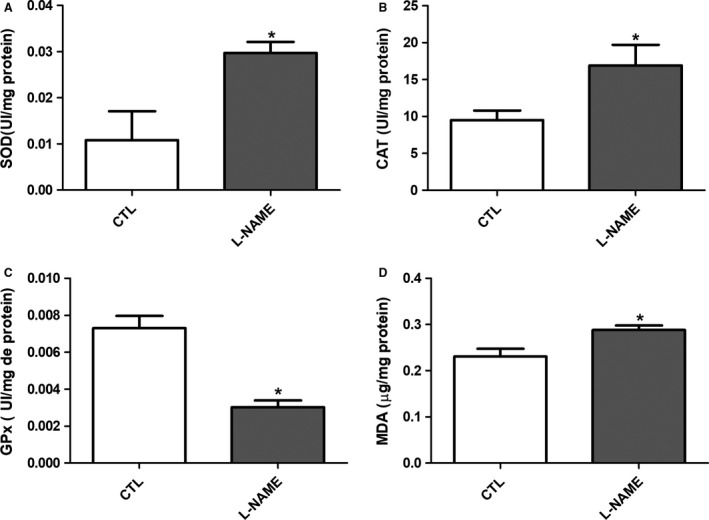
Systemic oxidative stress markers indicated by serum superoxide dismutase (SOD) (A), catalase (CAT) (B) and glutathione peroxidase (GPx) (C) activities and malondialdehyde (MDA) (D) in the CTL and L‐NAME‐treated group.*P < 0.05 compared with the CTL.

The values for serum SOD, CAT, and GPx activities are shown in Figure 6A–C, respectively. SOD and CAT activity were increased in the L‐NAME‐treated group compared with those in the CTL group. GPx activity was reduced in the L‐NAME‐treated group (Fig. 6C). MDA values were significantly increased in the L‐NAME‐treated group compared to the CTL group (Fig. 6D).

## Discussion

The major new findings of the present study were as follows: (1) a significant increase in rSNA and sSNA in L‐NAME‐treated rats; (2) a significant reduction in arterial baroreceptor reflex sensitivity to control rSNA but not sSNA; (3) a statistical correlation between plasma level reduction of NO and increased sSNA and MAP but not rSNA; (4) a significant increase in systemic oxidative stress in L‐NAME‐treated rats. Thus, in the present study, sympathoexcitation to the kidney and splanchnic regions was found, considering the hemodynamic repercussion of increased sympathetic actions in these two targets; this activation is probably an important mechanism triggering the increase in peripheral resistance and hypertension (Biancardi et al., 2007). Apparently, the plasma level of NO is a major determinant of splanchnic but not renal sympathoexcitation, suggesting that the sympathoexcitation, at least to the kidney, is probably independent of the reduction of NO systemically, local actions of NO, however, could be involved.

The hypertensive model of NO synthesis blockade has been linked to increased sympathetic vasomotor activity (Sakuma et al., 1992; Biancardi et al., 2007). However, there is still some controversy. Dos Santos and colleagues revealed that rats treated with L‐NAME for 2 or 14 days did not present significant alterations in rSNA despite the increased levels of arterial blood pressure (dos Santos et al., 2010). In the same direction, Ramchandra and colleagues did not observe increased rSNA in rabbits treated with L‐NAME for 7 days (Ramchandra et al., 2007). Some authors also suggest that SNA levels do not change with the inhibition of NO but that there may be an increase in the vascular response to a given level of SNA (Nase and Boegehold, 1996; Vials et al., 1997). Scrogin and co‐workers confirmed this result by verifying that the plasma catecholamine levels were not altered during acute inhibition of NO (Scrogin et al., 1998).

On the other hand, other studies have found that acute inhibition of NO (10 min or 3 h) promotes sympathoexcitation, which was more evident in baroreceptor denervated animals (Sakuma et al., 1992; Augustyniak et al., 2006). In addition, it has been demonstrated that renal denervation protects against the hypertension induced by L‐NAME (Matsuoka et al., 1994). In anesthetized rabbits with denervated baroreceptors, a pressor and sympathoexcitatory action of NO in the RVLM was reported suggesting that NO may activate sympathetic vasomotor activity (Hirooka et al, 1996).The different results obtained by these studies could be a consequence of either variations in NO bioavailability or a differential response induced by the inhibition of specific NO isoforms (endothelial, neuronal, inducible, and mitochondrial). On the other hand, selective nNOS inhibition by S‐methyl‐L‐thiocitrulline leads to activation of the vasomotor sympathetic nervous system via inhibition of NO in the brain (Shabeeh et al., 2017). In the present study, we found sympathoexcitation in kidney and splanchnic nerves; however, the sSNA but not rSNA activity was dependent on NO plasma levels. One important aspect to consider is that even after 1 week of L‐NAME treatment (20 mg/kg), the plasma NO was reduced by only 30%; thus, an incomplete inhibition of NO synthesis by L‐NAME was reached in the present study. Therefore, the large variation in the autonomic dysfunction and sympathetic vasomotor response to L‐NAME inhibition described in previous studies could be related to methodological issues, including the L‐NAME dose, time and method of treatment (gavage or diluted in the drinking water), the level of NO inhibition, and the sympathetic nerve recorded.

A possible mechanism leading to sympathoexcitation in response to NO inhibition is based on a previous study revealing that low concentrations of NO derived from nNOS or endothelial nitric oxide synthase (eNOS) promote changes in glutamatergic neurotransmission in the NTS and RVLM which lead to sympathoexcitation (Chan and Chan 2014). On the other hand, a high concentration of NO generated from iNOS potentiates γ‐aminobutyric acid (GABAergic) neurotransmission in the NTS and RVLM, resulting in sympathoinhibition (Chan and Chan 2014). Thus, one hypothesis is that changes in NO concentration in the brain are probably a major mechanism leading to sympathoexcitation and hypertension in L‐NAME‐treated rats. The RVLM is a major region leading to sympathoexcitation in L‐NAME‐treated rats considering that inhibition of this region leads to a reduction in blood pressure to a similar level observed after ganglionic blockade by hexamethonium in normotensive rats; this suggests that all hypertension in L‐NAME rats is determined by sympathetic drive (Bergamaschi et al., 1999).

Another possible mechanism leading to sympathoexcitation in L‐NAME‐treated rats is the activation of the renin‐angiotensin system in response to NO inhibition (Crowley, 2014). Angiotensin II is able to increase the oxidative stress activating enzyme, NADPH oxidase, which leads to the formation of intracellular superoxide anion (O_2_^−^). For instance, infusion of Ang II in rats induced hypertension and significantly increased the vascular formation of O_2_^−^. Interestingly, antioxidant treatment significantly reduced blood pressure in Ang II‐infused rats but not catecholamine‐induced hypertension (Laursen et al., 1997). Recently, our group showed that in L‐NAME‐treated rats, antioxidant treatment with green tea significantly reduced blood pressure, SNA, and oxidative stress markers (Garcia et al., 2017), suggesting a positive correlation between oxidative stress and sympathoexcitation in L‐NAME‐treated rats. In the present study, we also found a significant increase in oxidative stress estimated by the serum levels of MDA in L‐NAME group.

In conclusion, we found a significant increase in sympathetic vasomotor activity to the kidney and splanchnic territories, with a differential baroreceptor dysfunction as well as with a significant reduction in baroreflex sensitivity for renal but not for splanchnic nerve activity. However, sSNA, but not rSNA, is dependent on NO plasma level reduction, suggesting that the sympathoexcitation, at least to the kidney, is less dependent on circulating NO levels.

## Perspectives

Considering that sympathoexcitation is a major mechanism leading to hypertension, elucidating the actions of NO controlling the sympathetic vasomotor activity could improve our understanding regarding the onset and maintenance of hypertension and other diseases characterized by increased sympathetic activity, including heart failure. Increased oxidative stress and the actions of NO in the brain could be a new therapeutic target to overactivation of sympathetic vasomotor drive in pathophysiological conditions.

## Conflict of Interest

None declared.
